# Advancing evidence-based treatment of infectious diseases in children with real-world data: Opportunities and challenges

**DOI:** 10.3389/fphar.2022.1054688

**Published:** 2023-01-12

**Authors:** Kendra K. Radtke, Atul J. Butte

**Affiliations:** ^1^ Bakar Computational Health Science Institute, University of California, San Francisco, San Francisco, CA, United States; ^2^ Department of Pediatrics, University of California, San Francisco, San Francisco, CA, United States

**Keywords:** real-world data, pediatrics, children, infectious disease, drug safety, drug outcomes

## Abstract

There is an increased interest in utilizing real-world data (RWD) for pharmaceutical research and regulatory decision-making. The development and use of pediatric medicines could benefit greatly from real-world data studies given nearly half of drugs prescribed to children are “off-label”, meaning there is a lack of pediatric-specific evidence from controlled trials, while there is an abundance of data from routine clinical practice. Currently, the use of real-world data, such as data from electronic health records, is lacking in pediatric research, especially within infectious diseases. Here, we discuss opportunities and challenges for real-world data to generate evidence on the optimal treatment and management of infectious diseases in children.

## 1 Introduction

Children have been historically regarded as therapeutic orphans in drug development, resulting in few drugs with pediatric indications and a lack of evidence in drug labels approved by the United States Food and Drug Administration (FDA) (McCune, 2022). While there has been great improvement in pediatric labelling and evidence generation since the Best Pharmaceuticals for Children’s Act (BPCA) and Pediatric Research Equity Act (PREA), significant gaps remain, including long delays in pediatric approval compared to adults (8–10 years on average) (Beleck and Nachman, 2021).

One consequence of delayed, limited, or lacking pediatric evidence is the prescription of drugs for “off-label” use. Off-label use constitutes the prescription of a drug outside the prescribing information authorized in the drug label or package insert, including indication, age, dose, formulation, or administration route (COMMITTEE ON DRUGSNeville et al., 2014). Studies have found that 42%–73% of drug prescriptions in patients under 18 years were off-label, with the highest frequencies reported in neonates (Cuzzolin and Agostino, 2016; Hoon et al., 2019; Yackey et al., 2019; van der Zanden et al., 2022). However, off-label use does not mean off-evidence nor does it imply improper or contradicted use (COMMITTEE ON DRUGSNeville et al., 2014; Czaja et al., 2017). Highly prescribed drugs off-label may have strong evidence for safe and effective use, yet a formal application for regulatory authorization may not be sought (Czaja et al., 2017). In a recent study of the Dutch Pediatric Formulary, 42% of prescribed drug records were off-label, of which only 14% were supported by high-quality evidence and 37% by expert opinion (van der Zanden et al., 2022). This study confirms a lack of pediatric evidence in addition to off-label prescribing. Without pediatric evidence, the best dose, frequency, and duration for safety and effectiveness is unknown and under or over treatment are possible with potential consequences of adverse drug events and/or therapeutic failure.

To date, legislative and regulatory actions aimed to improve pediatric drug labelling have focused on incentivizing and improving clinical trials (McCune, 2022). In this article, we describe opportunities for real-world data (RWD) to contribute pediatric evidence towards the safe and effective use of medicines in children. We focus on anti-infectives as these are among the most common drugs prescribed to children (on or off-label) (Cuzzolin and Agostino, 2016; Hoon et al., 2019) and improper use may contribute to the emerging threat of antimicrobial resistance in pediatric infections (Romandini et al., 2021).

### 1.1 Real-world data available for pediatric infectious diseases research

RWD broadly describes any data generated from routine delivery of healthcare and/or relating to a patient’s health status (Food and Drug Administration, 2022a). RWD can come from various sources such as health insurance claims, electronic health records (EHRs), healthcare devices and wearables, and patient or disease registries (Rudrapatna and Butte, 2020). This article mainly focuses on EHRs as source of RWD for pediatric infectious disease research.

EHRs contain extensive data assets that can be leveraged in infectious diseases research (Table 1). Exposure to an anti-infective agent, either as a binary variable or composite of dose, frequency, and duration, can be determined from medication records. Inpatient medication administrations, which may be time- and date-stamped at administration in some EHR systems, are the most detailed source of drug exposure. Medical conditions and diagnoses from medical billing codes (e.g., ICD-10-CM) and/or measurement results can be used for cohort construction of a particular disease, as outcomes for adverse drug events or effectiveness, or to ascertain comorbidities for covariate analysis. Microbiological data collected as part of routine care can be used to characterize the infection and resistance within a patient, population, or system.

**TABLE 1:** EHR data available for pediatric infectious disease research.

Data asset	Potential uses	Case example
Medication records	Cohort construction	People prescribed azithromycin
	Drug exposure	Time- and date-stamped drug administrations
Immunization records	Cohort construction	People vaccinated against influenza
Measurements	Cohort construction	People with SARS-CoV-2 infection
	Outcome (safety)	Thrombocytopenia defined by low platelet count
	Outcome (pharmacology)	Vancomycin trough concentration
	Covariates	Elevated serum creatinine
Microbiological data	Cohort construction	People with methicillin resistant *Staphylococcus aureus*
	Pharmacodynamic endpoints	Minimum inhibitory concentration
Medical conditions	Cohort construction	People with HIV
	Covariates	Chronic kidney disease
	Outcome (effectiveness)	Incident *tuberculosis*
	Outcome (safety)	Prolonged QT syndrome
Encounters/visits	Outcome	Hospitalization event
Death records	Outcome	Mortality
Demographics	Covariates	Social determinants of health
Unstructured text data	Outcome	Extract subjective symptoms and time of resolution
	Other	Adherence assessment, clinical reasoning, suspected clinical findings
Radiological images	Development of AI/ML diagnostics	Lung imaging in pneumonia

Clinical ‘cure’ from infectious disease is challenging to ascertain with certainty in routine practice; thus, it is not a reasonable effectiveness outcome in EHR-based infectious disease research. One RWD study of six medical centers evaluated the outcome of clinical failure, defined as composite 30-day mortality, 30-day microbiological failure (infection reoccurrence with the same organism), and failure to resolve symptoms during therapy (Jorgensen et al., 2019). However, repeat microbiological cultures are not routine practice in the management of most infectious diseases. Symptoms may be documented in unstructured clinical notes, which could be utilized for research (if available), but this takes additional effort. Instead, indirect measures of effectiveness may be more appropriate, such as hospital readmission, length of hospital stay, mortality, or prescription of second line agent.

Quantitative drug measurements in plasma are an essential component of dose determination in pediatric drug development because absorption, distribution, metabolism, and elimination change as a child grows and develops (Kearns et al., 2003). These measurements are a standard component of clinical drug development programs (i.e., phase I and phase II studies), especially in pediatrics where, under certain conditions, efficacy can be extrapolated from adults to children with matching drug exposures (Food and Drug Administration, 2022b). Quantitative drug measurements may be available in EHR data systems, mainly from therapeutic drug monitoring (TDM) programs in place at the institution. Examples of anti-infective drugs in TDM programs include vancomycin and aminoglycosides (Wicha et al., 2021). Ad hoc drug levels may be ordered if a drug exhibits high inter-patient variability or a narrow therapeutic window.

### 1.2 Opportunities to generate real-world evidence in pediatric infectious diseases

Analyses with RWD have the potential to generate meaningful real-world evidence that can support regulatory decision-making and guide clinical practice. This impact was certainly demonstrated during the COVID-19 pandemic when RWD was utilized by research groups worldwide to generate evidence on disease progression, outcomes, and vaccine effectiveness and safety (Bennett et al., 2021; Li et al., 2021; Vashisht et al., 2021; Botton et al., 2022; Xie et al., 2022). Compared to this, utilization of RWD in pediatric infectious diseases research has been limited beyond the development or application of precision dosing tools for narrow therapeutic index drugs like vancomycin (Abdulla et al., 2021). But with the wide-spread adoption of electronic health systems, commitment to store these data for research in standardized formats, and advancements in computational power, there are new opportunities for RWD to support infectious diseases research in children. Some opportunities for research include.

- C*omparative safety and effectiveness*

Head-to-head comparison of pharmaceuticals in randomized controlled trials is rare beyond the investigational drug and an active control. Observational studies utilizing RWD such as EHRs have the potential to contribute evidence on comparative safety and effectiveness of drugs on the market typically used to treat a disease. Such evidence can be useful in informing treatment selection to optimize effectiveness and safety or identify patient subgroups that may benefit from one pharmaceutical intervention over another (Arterburn et al., 2018; Suchard et al., 2019). The study population, design, and statistical methodologies must be well-defined and rigorously developed to ensure findings are valid and unbiased (Sarri et al., 2022). Safety analysis will be limited to known adverse events where data (e.g., laboratory measurements) is routinely collected as part of therapeutic management and monitoring.

- *Studying antimicrobial treatment duration*

Current infectious diseases research supports shorter antibiotic treatment courses for acute infections than the historical standard (Spellberg et al., 2016). These conclusions are strongly driven by data from randomized clinical trials, including a recent study in children with community-acquired pneumonia (Spellberg et al., 2016; Williams et al., 2022). EHR data has the potential to assess similar questions on antimicrobial treatment duration permitted there is sufficient diversity in prescribed durations and appropriate measures are taken to account for confounding factors of treatment duration.

- *Pharmacology studies of dose-(concentration)-response*

The relationship between dose, concentration, and response are critical for informing optimal treatment of infectious diseases. Such analyses require a range of tested doses and/or concentrations for characterization. Quantitative drug measurements from e.g., therapeutic drug monitoring (TDM) would provide concentrations for population pharmacokinetic modelling or concentration-response analysis. Dose-response can also be evaluated with sufficient dose heterogeneity in the pediatric population. However, EHR data may contain homogenous dosing in the pediatric population (e.g., amoxicillin 22.5 mg/kg/dose twice daily). In this case, drug concentrations (measured or predicted from e.g., population pharmacokinetic models) can be used to inform dose-(concentration)-response along with other factors known to influence drug exposure such as age, body weight, or renal function. These analyses could identify pediatric subgroups at risk of suboptimal dosing.

- *Predicting antimicrobial resistance and Clinical Decision Support systems for antimicrobial prescribing*

Application of machine learning to EHR data has allowed the prediction of bacterial resistance with good accuracy (Yelin et al., 2019; Kadri et al., 2021; Lewin-Epstein et al., 2021). These algorithms can be developed into Clinical Decision Support systems to assist in appropriate empiric antibiotic therapy selection, for example.

- *Descriptive studies for hypothesis generation*

EHR data can be used for discovery or descriptive studies. For example, data from EHRs can be analyzed to understand the antimicrobial prescribing patterns in the pediatric population (e.g., demographics, doses and durations used, indications) or adverse drug outcomes with a particular high-risk drug or combination of drugs. These findings can be used learn where gaps exist in knowledge and to generate hypotheses that can be evaluated in future prospective studies.

### 1.3 Challenges to using real-world data in pediatric infectious disease research

Epidemiological and biostatistical challenges of using RWD for biomedical research has been previously discussed (Beaulieu Jones et al., 2020; Rudrapatna and Butte, 2020). Since EHR data are not collected for the primary purpose of research, studies will be limited by the availability and quality of the data, including accuracy and completeness. Data from hospital systems may not be connected to primary or specialty care systems and *vice versa*, resulting in a potentially incomplete patient profile. Since effective treatment of infectious diseases depends on host (immune), pharmacological, and pathogen factors, incomplete or inaccurate data on these factors could impact study findings. There is inherent uncertainty in the amount of drug a patient took in the outpatient setting. Infectious disease diagnosis may be based on clinical assessment alone; therefore, the pathogen, susceptibilities, and site of infection may be unknown. Furthermore, RWD pharmacology studies utilizing quantitative drug concentrations will likely be limited to drugs with an existing TDM program and in patients for which clinical need is present (e.g., risk of toxicity) since TDM is not widely utilized in the management of infectious diseases except for vancomycin and aminoglycosides (Wicha et al., 2021). Predicted drug concentrations from population pharmacokinetic models could be used *in lieu* of measured drug concentrations if such models exist.

## 2 Discussion

RWD are currently underutilized in pediatric infectious diseases research. While pediatric clinical trials will remain critical for pediatric drug development, especially for dose-finding pharmacokinetic studies and evaluating investigational drugs not yet approved for use, there is much potential for RWD to be leveraged in the post-approval or emergency use authorization period. These data can address key gaps in knowledge, circumventing the financial and recruitment challenges faced in pediatric trials. There are many opportunities for RWD to contribute to our understanding of optimal infectious diseases management in pediatrics as discussed in this article. Given some of the limitations in using RWD for research, studies will require careful consideration of the data’s fit for purpose. Study cohorts need to be thoughtfully and systematically constructed. Data should be adequately explored for accuracy and completeness and the appropriate statistical methods applied to control for potential bias when possible.

To better enable use of RWD in future pediatric infectious diseases research, enhanced data sharing along with adoption of common data structures is needed. This will enable larger, more diverse populations from multiple institutions to be studied more easily. Additionally, further development and reporting on data quality and standards of RWD assets and biostatistical methodology to handle incomplete or missing data will enhance the quality of evidence generated from the research. Investment from national and global research and regulatory agencies like the National Institutes of Health (NIH), World Health Organization (WHO), United States Food and Drug Administration (FDA), and European Medicines Agency (EMA) is needed to drive this field forward. An example of such investment is the newly established NIH Maternal and Pediatric Precision Therapeutics Hub (MPRINT), which “serves as a national resource for expertise in maternal and pediatric therapeutics to conduct and foster therapeutics-focused research in obstetrics, lactation, and pediatrics while enhancing inclusion of people with disabilities” and contains a real-world evidence core (mprint.org). With growing commitment from these agencies, enhanced RWD utilization in pediatric infectious diseases research will follow.

## Data Availability

The original contributions presented in the study are included in the article/supplementary material, further inquiries can be directed to the corresponding author.
